# Toward a performative epistemology of the archive: archival enactment as Rum futurity

**DOI:** 10.1007/s10502-025-09507-8

**Published:** 2025-09-05

**Authors:** Christina Banalopoulou

**Affiliations:** Department of Cultural and Environmental Heritage, University of Milan, Milan, Italy

**Keywords:** Rum minority, Turkey, Community archives, Performance ethnography, Performativity, Alternative futures

## Abstract

This article maps the alternative archives of the Rum community, those that do not fully align either with national mnemonics or the minority’s dominant archival politics. I demonstrate how the Rum minority performs and enacts its archives—what I define as archival enactment—in order to claim grassroots agency in its mnemonic preservation. Despite their sociopolitical and cultural significance and their important potential for the study of archival science, the Rum minority’s archival practices have been almost entirely ignored in the scholarship. Rum enacted archives call for a methodological shift from what the archive is—which is a definitory inquiry—to what the archive does and what worlds its transference constitutes—which is a question regarding archival performativity. Drawing upon rigorous multi-sited archival and ethnographic research, I show how the Rum minority performs its archives not just as documents of the past in need of interpretation but, most importantly, in the present as part of its envisioning and enacting of alternative futures. Blurring the boundaries between everyday performance, as expressed in familial and community settings, and artistic performance, as expressed in theatre, Rum archival enactment offers a great site for developing a dialogic practice of archival research that suspends the power differential between the archive and the researcher. I thus propose a performative epistemology for studying the archive that employs performance not only as archival content but also as transference and production.

December 13, 2023: I am attending the presentation of Stavros X. Giwltzoglou’s book *Ομογενειακή Παιδεία σε Κωνσταντινούπολη και Ίμβρο: Τα Ρωμαίικα Σχολεία στον 21*^*ο*^* Αιώνα* [Rum Minority Education in Istanbul and Imvros: The Rum Minority Schools in the 21st Century] at the *Πνευματικό Κέντρο Κωνσταντινουπολιτών* [Cultural Center of Istanbulites] in Athens. There are a series of speakers positioned on stage consisting of public figures of the Rum community in Turkey as well as its diasporas in Greece. To open the event, one of its co-organizers, acknowledges the tensions that the book has raised within the community regarding the limits of archival research. After a few speakers presented their mostly positive thoughts on Giwltzoglou’s project, the talk’s coordinator went to the podium and read a script composed and sent by members of the community in Turkey, mostly educators and school administrators, who were critical of the author’s archival methods. Part of the enacted script included the following: “I disagree with the author’s violation of the personal data of our educators […] his breach of confidentiality [and] intentional lack of protection of our educators jeopardizing the function of our school” (intv.gr 2023, 52:44–53:17)^1^. The Rum community’s enactment of a collectively scripted text as a minoritarian intervention regarding the porousness of the boundaries between the archival private and the archival public offers valuable insights into a societally informed archival methodology that suspends the power differential between the researcher and the researched.

Preceding the formation of the nation-state, the term Rum [Roman/Ρωμηός]^2^ refers to the ethnoreligious Christian Orthodox communities of the Byzantine Empire. In the Ottoman Empire, the Rum communities were organized into the Rum millet—a self-governed non-Muslim Greek Orthodox community led by the Patriarch and answerable to the Ottoman Sultan (Alexandris 1992, pp 21–24). Once Turkey was established as a Republic under the Treaty of Lausanne in 1923, the Rum community was internationally recognized as an ethnoreligious minority associated with the Greek nation, which often shares antagonistic geopolitical interests with Turkey. Caught in between competing nationalisms and the tensions between religion and secularism, the minority faced a series of expulsions and demographic engineering practices throughout the twentieth century. To this day, the minority’s interests do not always fully align with Greece, Turkey, or the revivalist afterlives of the Ottoman Empire, and its survival is still characterized by precarity.

Due to its precarious state, the members of the community are often hesitant to share their archives with researchers. Attending the event helped me recall, both from my Rum family’s histories—my family left Istanbul, Turkey in 1974–75 as part of the last peak of the “soft expulsions” that followed the tensions in Cyprus—and my research on Rum theatre and performance, that performing and enacting the archive plays a key role in the community’s efforts to reclaim grassroots agency in mnemonic preservation—what I define as archival enactment. After migrating from Turkey to Greece, my family transferred soil and basil and geranium seeds from their country house in Burgazada/Antigone and planted them in the garden of their new home in Athens to recreate their experiences from their island so that their memories from Turkey were transferred to the next generations. As both a diasporic member of the community and a researcher of its theatre worlds, I had the opportunity to witness and participate in grassroots practices of archival transference and preservation that utilize the permeable borders between everyday performance, as expressed in the case of my family and the broader community, and artistic performance, as expressed in theatre. During my participant observation, many members of the community shared their theatre archives with me. As they did so, they enacted each page, reading it aloud to me and sharing memories, stories, and feelings connected to the archival document. Their enactment of the archive was an integral part of its transference and the community’s mnemonic agency.

In this essay, I investigate the epistemological and methodological implications of the Rum community’s archival enactment practices that intersect three layers of archival praxis: family, community, and theatre. I show how performance, both everyday and artistic, plays a significant role in the ways the community claims participatory agency over the afterlives of its archives and the conditions of its archival resilience. For Angelos Dalachanis and Agamemnon Tselikas, “the often-fragile position of the Christian communities in an unstable political and geopolitical context, along with their almost existential suspicion of each other, prevents them from sharing their archival material, which is often extremely rich and quite often unexamined” (2018, pp 132). While archival closedness can impede research’s critical capacity to intervene and cultivate social change, co-performing the archive can also lay the grounds for the development of research methodologies that open up issues of archival access to active and ongoing communal investment both from the end of the involved community and the researcher. By addressing the involved community’s mnemonic interventions through performance as integral to the archival research process, archival science can expand its informational focus to include the acknowledgment of its constitutional capacities. Thus, rather than primarily permitting or impeding informational access, archival skepticism or openness becomes an integral part of archival knowledge production.

Rather than solely approaching the archive as an object of study, archival enactment recognizes archival research’s capacity to contribute directly to the lives of the involved populations. Far from neglecting the significance of their role, the Rum community invites the researcher to conduct a shift of their epistemological focus from what the archive is—which is a definitory inquiry—to what the archive does and what worlds its transference constitutes—which is a question regarding archival performativity. For Eric Ketelaar, “records have agency because they can accomplish something, make a difference in status before and after” (2017, pp 253). Rum enacted archives are not just documents of the past in need of objective interpretation. On the contrary, the Rum minority co-performs them in the present as part of its minoritarian envisioning of alternative futures. After conducting a brief literature review on the intersections between the performative and the archival, I contextualize my utilization of a performance-based ethnographic framework for studying Rum archives by grounding them in the community’s histories.

## Archive’s performative twists and turns

The performative turn in the archive has been part of archival science’s increasing investment in examining more how archives perform, the power relations they reproduce, and the solidarity systems they cultivate and less in what content they entail (Cook 2013). On a level of epistemology, archival studies have been exploring performance’s contribution to archival methodologies that shape more horizontal and participatory relations between the researcher and their body of research (Cook and Schwartz 2002). Archival studies’ interest in performance is inseparable from its incorporation of methodologies from other disciplines, such as cultural studies and critical ethnography as a response to postcolonial studies’ criticism of the archive as a site of power that excludes minoritarian and underserved populations (Papaelias 2005; Stoler 2010). Current debates on archival practice involve a rich and growing scholarship that widens the range of what constitutes an archive through the performative (Altınay and Jokić 2020, 2021; Altınay 2022) and the sensorial (Cvetkovich 2003) and expands the discipline’s methodologies (Gilliland and Halilovich 2017; Rokay 2021; Punathil 2021) by incorporating cultural, performance, queer, and critical race and postcolonial theory (Summers 2015; Tortorici 2022; Shomali 2023). Critical archival studies have demonstrated that community, unorthodox, and personal archives disrupt the hegemonic relations that institutional archives perpetuate (Cook and Schwartz 2002; Azoulay 2019) and that the study of grassroots archives involves not so much the deciphering of the past but the dynamic envisioning and enacting of the future (Abbas and Abou-Rahme 2013; Hochberg 2021).

Archival studies’ interest in performance coincides with performance studies’ growing interest in the archive. Performance studies understands the archive as a dynamic process during which the lived ephemeral introduces the remaining textual to what Soyini Madison defines as “the possibilities of performance” (2019, pp 171). Any embodied, sensed, and performed acts of mnemonic transference—what Diana Taylor calls the *repertoire*—entrust the archive to other spheres, nexuses, and temporalities (2003). Theatre and performance archive the non-repeatable aspects of a repeatable script (Carlson 2001), and rather than occurring due to archival failure, reenactment activates the potentialities of an archive (Lepecki 2010, pp 31). However, despite these important advances regarding frameworks that address the interconnections between the informational and the constitutional aspects of the archive as well as its textual and performed dimensions, discourse tends to ignore how performance intervenes in the afterlives of the scripted archival. The ways in which the Rum community employs both everyday and artistic theatricality to transfer its family, community, and theatre archives offer a great platform for investigating minoritarian reconfigurations of the hegemonic distinction between archival text and performance.

Rum archival practices’ reliance on enactment emerges from the community’s negotiations of its minoritization while also eluding the competing tensions between Turkish and Greek nationalisms as well as secularism and the afterlives of the ethnoreligiously diverse Ottoman Empire. The Rum community became a minority under the Treaty of Lausanne—the international agreement that established Turkey as a nation-state in 1923. Reflecting the ethnic-cleansing logics and the homogenizing practices of the nationalization projects of Turkey and Greece, the Treaty included the implementation of the compulsory Greco-Turkish population exchange, which, neglecting ethnicity or language, involved the forced migration of more than 1,2 million Greek Orthodox people from Turkey to Greece and 355,000–400,000 Muslims from Greece to Turkey solely on the basis on religion. The Rum Orthodox populations in Istanbul and the Muslim populations in Thrace were exempted from the exchange. The Treaty’s principle of reciprocity rendered the survival of these two communities interdependent. Far from merely relying on mutuality, however, the Treaty’s reciprocal aspects allow for more negative employments of the principle (Akgönül 2008). Within these frames of potential retaliation, the minorities who stayed behind are often treated less as citizens and more as instruments that serve the advancement of often competing diplomatic and geopolitical interests. Therefore, the Rum minority often designs its archival practices, especially the ones that relate to its family histories and community-building practices, including theatre, to focus more on the everyday and less on the institutional.

The Treaty’s retaliatory logics were not the only factor that rendered the Rum minority precarious. As Meropi Anastasiadou and Paul Dumont’s work indicates, under the Treaty of Lausanne, the Rum minority’s survival depends on maintaining their ethnoreligious identity, which is, by default, contradictory to Turkey’s projects of secularization and national homogenization (2010, pp 34–38). Due to nationalism’s tensions with ethnoreligious diversity and the afterlives of Lausanne, the minority faced a series of expulsions both “soft” and compulsory. For Alexis Alexandris, in addition to the Treaty’s ongoing manifestations, the causes of these exoduses also date back to the previous century’s financial divisions between the Empire’s Muslim and non-Muslim communities (1992, pp 31). Regardless of the temporal frames and the factors that contributed to the demographic engineering practices that were employed throughout the twentieth century, however, as Alexandris argues, “The Greek in Republican Turkey lived simultaneously at two levels of experience. On the one hand, he conducted his everyday life as a Turkish citizen and loyally observed the responsibilities that his citizenship entailed. But, there was another level of experience closely connected with his traditional ethnic Greek/Rum identity and customs, which he was not prepared to abandon” (pp 296). Archival enactment serves as a site for the community to preserve its archives outside of these schizoid frames and institutionally defined memory politics.

In addition to eluding the competing tensions between the national and the minoritarian mnemonic, as well as religion and secularism, enactment functions as a tool for the minority to transfer its lived archives, memories, and feelings throughout its migration. As Ilay Romain Örs’s rigorous urban ethnographic research on Rum diasporas demonstrates, “the history of the Rum Polites community is intrinsically linked to that of the city” (2018, pp 5). Thus, recreating their relation with the city’s topographies is one of the main practices that help the Rums of the diaspora feel that they are not extinct. In spite of their sociopolitical and cultural significance, however, the Rum archival practices that either question or simply remain indifferent to national and minoritarian archives are relatively ignored in the literature. Scholarship has shown how the community’s histories are interconnected to its demographic changes (Αναστασιάδου και Ντυμόν 2010), its exoduses (Alexandris 1992), urban landscapes (Örs 2018), minoritarian institutions (Κάννερ 2004; Φραγκόπουλος 2009), connections to Ottoman and Turkish historiographical frameworks (Philliou 2022), and its elite class who generated from above sentiments of nationalism within the lower strata of the Rum community (Kamouzis 2021). While the histories of the Rum community have been significantly studied, the potential for examining Rum archives that do not fully align with the minority’s institutionalized mnemonics and that require interdisciplinary archival research combined with ethnographic methodologies remains understudied. The Rum minority’s family and community archives that are transferred in the act of both everyday and theatricalized performance offer a great vantage point for examining other aspects of Rumness that align neither with the projects of Greek and Turkish nationalisms nor with imperial revivalisms.

## Performing the archive, researching the archival

Rum archival enactments do not merely transfer information but, importantly, recreate and generate experiences, emotions, senses, and desires. Methodologically, thus, they require Ann Laura Stoler’s definition of the archives “not as sites of knowledge retrieval, but of knowledge production” that call for an “ethnographic” and not an “extractive” approach (2002, pp 87). Adding to Stoler’s suggestion to study archives less as preset knowledge and more as ethnographic sites of reality production, including the sensorial, I argue that due to their emphasis on enactment and performance, Rum archives can be better understood through the tool of performance ethnography which employs performance as a research method and a way of knowing. As an ethnography both *of* and *through* performance, *performance ethnography* can address enacted archival transference as part of the archive and examine the constitutive aspects of Rum family, community, and theatre archives primarily through the temporal lens of collectively imagined and enacted futures. For Dwight Conquergood, in performance ethnography, the ethnographer does not simply document or examine the field. On the contrary, they engage in a “dialogical performance” as a way of doing research that “instead of speaking about [people], one speaks to and with them” (2013, pp 77). As immersive performances of archives, Rum archival enactments invite the researcher to “co-perform” the archive and embrace what Conquergood defines as “co-performative witnessing” (37).

Co-performative witnessing has been central to my research. I have conducted multi-sited archival and ethnographic research in Turkey and two of the most popular destinations of the Rum diaspora: Greece and the USA. During my participant observation, which included attending events, talks, shows, celebrations, and primarily Rum informal gatherings at houses, my interlocutors invited me to co-perform their archives with them as part of the sharing process. Especially at the gatherings, I participated in archival exchanges that utilized both everyday and theatricalized performance as a medium of transference. Rather than merely being treated as documents of the past, the shared photographs, artifacts, correspondence, playbills, and diaries inspired the reenactment of memories, scenes, and experiences both from everyday life and theatre for transgenerational relations to be cultivated as part of the minority’s collective visions and actualization of resilience and futurity. The domestic space of Rum living rooms was often transformed into a stage for stories to be shared through their enactment and my role as both a researcher and a member of the community was integral to these theatricalized processes of archival transference.

My archival research also included numerous institutions, such as the Library of the Ecumenical Patriarchate of Constantinople in Istanbul, the Sismanogleio Library, the Frangopoulos Library, and the Atatürk Library in Istanbul, Turkey, the Asia Minor Studies Center, the Library of the Hellenic Parliament, and the Eptalofos Library in Athens, Greece, and the Library of Congress, in Washington, DC, USA. The majority of the documents that I studied at these sites involved published original dramatic texts, newspaper articles, magazines, periodicals, official photographs, and correspondence. While access to these sites involved formalized application and approval processes, my ethnographic research, fieldwork, and participant observation allowed me to access community, unorthodox, personal, and hidden archives. These archives included playbills, diaries, personal notes and correspondence, production photographs, stage directions, and targeted newspaper articles about specific performances that I was unable to find in the library collections since they had either been lost, destroyed in fires, or even thrown away due to limited space.

Far from solely allowing me to access archives that are otherwise private, combining participant observation with archival research allowed me to witness the community’s agency over its archives. My identity as a member of the Rum diaspora, familiarity with the Rum dialect, and ability to speak Greek and Turkish helped me get invited to informal community gatherings. In many cases, my family’s social and professional networks introduced me to more members of the Rum community interested in sharing their stories and archives on the community’s theatre and performance practices. Even though my research is not strictly autoethnographic, a methodology that involves “the documentation of an individual life as research” (Miller and Taylor 2006, pp 170), it borrows tools from autoethnography. It intersects the personal, empirical, and epistemological to interact with the everyday archival. My research inquiry addressed less the content of the archives and more the ways in which both everyday and theatricalized performance transferred archives as sites of knowledge production during the community’s demographic decline and both directly and indirectly forced migration.

I have conducted 12 semi-structured anonymized interviews in Istanbul, Turkey, 7 semi-structured anonymized interviews in Athens, Greece, and 3 semi-structured anonymized interviews in the USA, including Washington, DC where the community maintains the Hellenic Society of Constantinopolitans, Greater DC Area. Even though the knowledge documented during these interviews is of significant value, the information regarding the community’s ways of transferring their archives with me as both a researcher and a diasporic member of the community is of equal importance. Because Rum theatre is practiced by and for the community, the majority of my interviewers ended up having some relation to theatre either as practitioners, theatre-goers, playwrights, community organizers, or teachers and educators who incorporate theatre practices in their pedagogy. In all these moments, the permeable membrane between the enduring textuality of the printed documents and the ephemeral liveliness of theatre and performance opened up the private sphere of personal and family archives to the public domain of community archives. Rather than solely being self-referential, the personal aligns with what Soyini Madison defines as a “composite of interpenetrating and polyvocal experiences, intents, and desires within itself and with Others” (2006, pp 320). As performance becomes the connective tissue between the archival personal, familial, communal, and theatrical, what remains cannot solely be traced in the written archive’s textuality but also in its enactment. Since in the backdrop of the community’s demographic decline, many of its members, in Meropi Anastasiadou and Paul Dumont’s terms, “refuse to be just a memory and-without rejecting the past-are trying to look towards the future” (2010, pp 16–17), the liveness of archival enactment infuses presence and future into the archived past. In the act of its performance, the archive remains through what Rebecca Schneider defines as performative logics of not “that which disappears (as the archive expects), but as both the *act* of remaining and a means of re-appearance” and that understand that “we are almost immediately forced to admit that remains do not have to be isolated to the document” (2011, pp 101). The next section examines how the Rum minority enacts its family and community archives in order to transfer them.

## Enacting the everyday, archiving the familial

When my family migrated from Turkey to Greece during the Cyprus events in 1974, they were unable to transfer their material belongings and personal archives, such as documents and photographs. However, they did transfer soil as well as basil and geranium seeds from the “island,” as many Rums refer to Burgazada/Antigone. They planted these seeds at their new home in Athens, enabling them to gather with other diasporic Rums to “smell the island’s aromas, sense its auras, and feel *as if* they are there” (interview with Rum family member who left Turkey in 1975, April 26, 2023). Most Sundays of my childhood involved other members of the Rum diaspora coming over to our home for tea in order to smell our plants and involve me in their collective recreation of their experiences from the island so that their memories were transferred to the next generations. In addition to playing a significant role in the Rum diaspora’s practices of reconnecting with each other in the backdrop of migration, the transported soil and seeds transferred senses, such as sights and smells, in the act of recreating them for future generations to embody. As my grandfather used to tell me while I was watering and maintaining the plants in our garden in Athens, “These are more important than pictures. We wanted you to know how it feels like to be on the island.”

Suspending our disbelief regarding our surroundings, the ones who participated in these gatherings, including myself, *as if* we were on the island, transferred sensorial information about the community’s lived experiences through performing the daily act of coming together. The planted and flourished basil and geranium were part of the material cultures involved in these aestheticized practices of recollecting a scattered community. The archival researcher thus needs to broaden "understandings of the archive through challenging distinctions between contexts and texts, texts and archives, and archives and bodies” (Edmondson 2023, pp vii). Especially for the Rums who left Turkey due to the twentieth century’s migratory expulsions, enactments of futurity relied on sensorial encounters that involved the dynamic somatization of transferred material culture.

In the case of my family’s archives, the impossibility of archival transference shaped the possibility of sensorial transference through enactment and performance. The basil and geranium were utilized and felt as archives of sensation that could be activated in the future by the dynamic interplay between materiality and collective experience. They functioned as what Ann Cvetkovich defines as an “archive of feelings […] as repositories of feelings and emotions […] that demands an unusual archive, whose materials, in pointing to trauma’s ephemerality, are themselves frequently ephemeral” (2003, pp 7). Far from being widely and immediately recognized as archives, however, the soil and seeds’ archival function remained imperceptible unless intentionally “invoked in the performance allowing the archive to speak” (Alexander 2012, pp 277). Rum diaspora’s embodied interactions with the materiality of the transferred soil allow for the performative potentialities of the material aspect of the archive to be studied and collectively explored.

While national borders and the urgency of migration rendered other types of archival transference impossible, the elusiveness of the soil and seeds regarding their archival function that can be potentially activated by and through performance allowed for a wider range of mobility and transferability. Therefore, rather than engaging with the archive as institutionally and nationally gatekept knowledge, the community’s sensorial archives invite the researcher to approach archival making as “a living record that constantly changes because it's constituted between bodies, in movement, in the transnational” (Shomali 2023, pp 2). Moving the archive involves multiple layers and directionalities of transference. The Rum community’s sensorial archives activated and transferred through performance did not solely achieve to, in Anne J. Gilliand and Hariz Halilovich’s terms,“(re)create a sense of ‘home away from home’ in often alien, sociocultural, physical, and bureaucratic environments” (2017, p. 80) but also reestablished a sense of home in the places the minority had to leave left behind. Performance, thus, as a method and an object of research, reinvents the archive even in the backdrop of archival loss or entrenchment.

Archival access at one of Istanbul’s Rum minority schools, which one of my family members attended as a child, was restricted. However, one of the school’s teachers and administrators took me on a very long walking tour around the building and invited me to engage with the space as a student would, embodying my family’s feelings and experiences both as a researcher and a diasporic member of the community. One of my family’s close friends and classmates currently living in Istanbul accompanied me on this process of co-performing Rum studenthood. We entered the school’s classrooms, sat at desks, went through the libraries’ books, and pretended to return to the classrooms after intermission. Some of the school’s teachers participated in the performance by writing sentences on the board and ringing the classroom bell. Others offered to record videos and take pictures of our make-believe performance so that I could share them with the member of my family who attended the school but was unable to join us since they no longer live in Istanbul and have not visited the school since they left in the 70 s. We all created a moving and sharable sensorial archive of experiences that documented the ephemeral reenactments of an imagined past.

Co-creating an archive of imagined experiences of past Rum studenthood in the act of performing in the present with the intentionality of sharing and reenacting it in the future gains new meanings when contextualized within the current frames of Rum minority education. The Rum minority has been shrinking since 1923. While in the early twentieth century, the Rum population was approximately 160,000, currently, the Rums in Istanbul number roughly 2–4000 (Αναστασιάδου και Ντυμόν, pp 26–27). The community’s demographic decline has affected the Rum school’s enrollment, which remains very low. Rather than merely generating feelings of nostalgia tied to the past, the moving archive of Rum studenthood shared in the present and future through its enactment functioned as the affective connective tissue that holds together the desires, temporalities, and connections that migration scatters. It established a participatory sensorial that, far from reminiscing past experiences, *re-members* them in the present. For Yannis Hamilakis, collective re-membering relates to the active recollection of a community that has been scattered (2010). When I shared this particular archive of senses of Rum studenthood with my relative, they told me that through these types of archives, “another Rumness” could be preserved (anonymized interview with a Rum family member who left Turkey in 1975). Rumness, just like other types of -ness, becomes “a relational quality or subject position rather than a fixed, essential definition open to authentication” (Shomali 2023, pp 6). My relative’s reference to an alternative notion of Rumness indicates a politics of Rum belonging that, rather than relying on institutional definitions of the minority, places emphasis on the community’s lived everyday.

The community’s archival practices are often mediated by the personal as part of its members' agency over Rum mnemonic preservation. In Athens, the Eptalofos Library in Kallithea, founded by the Rum Nikos Kürkçoğlu, who migrated from Turkey to Greece is one of the largest and most diverse Rum archival collections in Greece. In addition to published books, newspapers, magazines, encyclopedias, and manuscripts both in Greek and Turkish, it involves a wide range of Rum personal archives and library collections. Kürkçoğlu developed Eptalofos by traveling from Greece to Turkey annually, sometimes two times within the same year, and transferring, upon their family’s consent, the personal archives and collections of any deceased Rums. He also transferred numerous books that he purchased at various bookstores, including Turkish thrift stores.^3^ Eptalofos is the product of Kürkçoğlu’s active recollection and transference of the community’s mnemonic and cultural production. However, far from defining Rumness solely in relation to the mnemonic practices of the community, as he stated in the speech that he gave as part of the *Εταιρεία Μελέτης της Καθ’ Ημάς Ανατολής* [Society for the Study of the Near East] talk series on «Τόποι και Πρόσωπα της Πόλης» [Places and People of the City] “the original approach of Eptalofos has to do with its vast materials on the Greco-Turkish relations so that it can serve researchers of both angles.” In the same speech, Kürkçoğlu contextualizes the making of Eptalofos within his and his family’s personal histories, their life in Istanbul, and their migration processes. When I visited the library for the first time in August of 2023, the Eptalofos staff printed and shared this particular speech of his with me so that I became familiar with the library’s histories, which were interconnected to his own.

Dissociating the personal, which often intervenes through enactment and performance, from community archives prohibits many of the minority’s members from sharing their personal and private archives with the community’s institutions. Rum archival skepticism resonates with Cook’s fourth archival paradigm of “community archiving,” according to which “many community archives are distinctly uneasy about turning their archives over to state or other archives which represent (and are sponsored by) governments or other institutions of power that previously excluded them as unimportant, or, worse, in some cases actively discriminated against and persecuted members of these communities” (2013, pp 114). When I asked one of my interviewees why they do not hand over their archives to one of the community’s institutions in spite of the institutions' significant digitization efforts, they responded, “Because their essence will be lost” (anonymized interview, September 19, 2023). Drawing upon Walter Benjamin’s argument regarding the loss of art’s aura as proximity between the subject and the object in democratized contexts, Gil Z. Hochberg argues that the archive’s democratization—to the extent that it is rendered open to the public—also makes it “less auratic” (2021, pp 12). By repositioning the personal and the communal back to the archival through performance, the Rum minority redefines the aura of the archive on the basis of minoritarian participation in mnemonic preservation. Rather than implying the loss of essence in terms of authenticity, this particular quote reclaimed the Rum minority’s dynamic contribution in its archival afterlives.

## Moving theatre archives as future encounters

Enactment plays a crucial role in the minority’s archives that not only challenge or counter dominant and popular archival practices but, most importantly, exceed them. On March 5, 1963—a year before the 1964 compulsory expulsions of the Rum populations who also maintained Greek citizenship—the members of the Φίλοι της Τέχνης [Sanat Sevenler/ Friends of the Art] theatre company staged *Ο Άρχοντας του Κόσμου* [Dünyanın Hakimi/The Master of the World] at the Yeni Tiyatro [New Theatre] by the Greek playwright Timos Moraitinis and directed by the Rum Alkis Kronis. The play was a philosophical satire that dealt with the battle between good and evil. In the show’s playbill, the group writes, “The Friends of the Art theatre collective was created, as a core, many years ago. It worked silently and anonymously, renewing and complementing its constituents by including members from different neighborhoods and professional backgrounds who have one thing in common: their love for the theatre. This year, we thought for the first time to officially name ourselves the Friends of the Art so that we render the goal of our efforts more visible.” As opposed to the dominant tendencies of the political economies of Rum theatre, according to which each production was linked to a specific Rum parish, the Friends of the Art toured from parish to parish. For each show, members of the theatre collective collaborated with members of the community, enhancing the group’s community-building practices. The production of *The Master of the World*, for instance, was a collaboration between the Friends of the Art and the Pera Club. As a result, the group’s archives were nomadic and primarily administered by its members through their theatrical encounters since they did not officially belong to any of the minority’s institutions.

Reflecting how the group’s archival nomadism runs through the Rum community, on March 8, 1963,^4^ the Rum newspaper *Embros* published an article dedicated to a souvenir that the Pera Club gave to the Friends of the Art after their production of *The Master of the World.* As the article’s author, A. Fandris, writes, the souvenir, which was also accompanied by flowers, was a way for the members of the club to “express their gratitude.” While “the flowers wither [and] the standing ovations last for only a couple of seconds,” the souvenir is the “only thing that remains” (2). Far from solely framing the souvenir’s performativity in terms of temporal unchangeability, the author makes the case that this particular mnemonic tool “acquires more content with the passing of time” and “renders the past timely.” He describes its function as a “pluralization of the past […] that becomes the connective tissue between the past and the present,” reactivates the “sensorial” mnemonic, and “resituates all the amateur actors on the theatrical scene—this time through imagination— in order to reenact [past performances]” (2). As an object into which the community inscribed archival performativity—the capacity to function as an archive—the souvenir reenacts the past in order to activate embodied and sensorial mneme and to constitute presence and futurity.

Approaching the souvenir as an archive opens up the—often gatekept—archival institutional to the archival commons. As Rüstem Ertuğ Altınay’s work demonstrates, especially during the first 50 years of the Turkish Republic, acts that turned private archives into a “public repository” through experimenting with various genres that blur fiction with historiographical writing unsettled the dominancy of the nation-state’s official memory politics (2015, pp 95). Thus, the archival researcher is invited to conduct an “expansion of the archive from a relatively hegemonic and centralized site of power with limited access and great supervision to a reality in which even private photos or collections of ephemeral objects ‘count’ as an archive” (Hochberg 2021, pp 12). The studying of the souvenir as an artifact that, depending on its use, entails archival performativity, expands archival science’s focus on the cognitive and interpretative to also include the sensorial. The Rum community orients the souvenir’s archival performativity around its capacity to enact Rum futurity, sensorial encounters, and relations.

In the context of the Rum expulsions, both “soft” and compulsory, and the demographic decline of the Rum population in Turkey throughout the twentieth century, the enactment of future Rum gatherings gains important meanings about the community’s resilience. After wondering, “How did we end up so scattered? How did we become so dispersed?” Fandris frames the souvenir as the link between the past, the presence, and the future that entails the capacity to reenact past and new feelings, memories, and relations (2). As opposed to other types of official archives that remain tied to the border politics of nationalism, archival artifacts become easily nomadic in the light of directly or indirectly forced displacement. Through their performative acts of invoking the archive, the Rum community claims their right to share, perform, and transform their mnemonic repositories and to prioritize archiving as a collective praxis of their sensorial commons. The community’s future encounters are imagined on the basis of theatrical praxis and its archives.

Throughout my ethnographic research, while sharing their archives, stories, and experiences, many of the participants rendered the embodiment of this or similar material artifacts as an inseparable part of the process of archival transference. Especially props or objects that were shared as souvenirs among the community during or after their shows in Istanbul, such as rings, played a crucial role in the transmission and transformation of the community’s sensorial archives. During many of my conversations with Rum theatre practitioners in Turkey and Greece, they put on these objects while they were sharing their archives and enacting their memories and stories to establish a participatory sensorial, and, far from reminiscing it as a past experience, to recreate it in the present. For many of them, recreating their experiences made them feel that the community has a future: “I feel like what we leave behind and lived in the past will have a future” (anonymized interview with Rum theatre-goer, April 16, 2024). As a moving archival artifact, the souvenir entails the capacity to generate Rum belonging across spatial and temporal contexts.

I recently gave a public talk on Rum theatre and performance at the Hellenic Society of Constantinopolitans in Washington, DC. The participants consisted of first- and second-generation diasporic Rums who migrated to the USA during the twentieth century’s expulsions. During the talk, they interacted with some of the Rum theatre archives that I had collected during my research and carried to the USA in order to share with them. On this and other similar occasions, the participants had the opportunity to skim through Rum theatre playbills, production photos, and newspaper articles. Many of them were able to find their or their relatives’ names on the playbills and the attached advertisements or to recognize themselves and their friends in the shared photographs. One of the participants remembered that they had designed the frame of one of the theatre posters. Since Rum theatre has mostly been a practice by and for the community, its moving archives, both in terms of mobility and sensorial activation, served as a site for the performative and embodied production and transmission of knowledge across time and space.

Rum theatre archives often enact modes of Rum belonging that are often indifferent to institutional definitions of Rumness. As dynamic repositories that cultivate future encounters while interconnected to the community’s migratory flows, Rum theatre archives are on the move, and they are organized around what Hochberg describes as an act that “celebrates the mundane” (2021, p. 6). Gathering around transferred soil and seeds, reenacting Rum studenthood, and recreating scenes from its theatre archives across borders, the Rum community utilizes enactment in order to archive and celebrate the everyday. Resonating with José Esteban Muñoz’s work on “ephemera *as* evidence,” Rum theatre archives embrace enacted ephemerality as “modes of textuality and narrativity […] that remain after a performance, a kind of evidence of what has transpired but certainly not the thing itself” (1996, pp 10). Intersecting the empirical with the epistemological, studying the archive’s ephemeral aspects does not neglect its materiality. On the contrary, “it reformulates and expands our understandings of materiality” (10). Rum archives remain through the relations, futurity, and knowledge their ephemerality constitutes. Besides, the archival practices of ethnic minorities share tensions with the ways in which ethnic archives are designed and curated nationally (Dominique 2014). Therefore, the survival of grassroots ethnic archives often relies on ephemerality. Especially in contemporary Turkey, the tensions between archiving ethnicity and archiving nationalism occur either because of the ethnic groups’ systemic and discriminatory inclusion by exclusion that serves the homogeneity of the nation-state or because the nation often instrumentalizes the involved ethnic groups in order to justify its neocolonial, imperial, and neoliberal politics.

The epistemological incorporation of the porous borders between the institutionalized archival and the archival every day can shed light on how Rum archival practices question the hegemonic relations of revivalist imperialism and nationalism. As the work of Alexis Rappas shows, far from establishing abrupt discontinuities between Ottoman and post-Ottoman modes of governance, the European rule of post-Ottoman provinces involved more complicated governing tactics (2022). On a level of archival politics, questioning the interstitial spaces between colonial and (post)colonial governance mechanisms involves not only cognitive but also performative and sensorial ways of archiving and studying the archive. The epistemological rejection of colonial and (post-)colonial archival paradigms require what Zeb Tortorici describes as decolonial archival imaginaries that “merge ideas of how both decolonial praxis and archival theory can, in a gesture toward Benedict Anderson among others, be productively redeployed through the concept of the imaginary” (2022, pp 67). The methodological transition from the archival factual to the archival enacted, especially because of Rum archival enactment’s employment of the imaginative around alternative futures and politics of belonging that focus on the everyday could contribute to archival research and practices that dissociate the archival from the colonial.

## Conclusion

A growing body of research applies critical ethnography as an archival tool to challenge the archival alienation of minorities from mainstream institutional archives (Rokay 2021; Punathil 2021). Rum archival enactments demonstrate how minorities are often indifferent to archival practices primarily led by various institutions, from state archives to non-profit organizations and archives curated by the community’s institutional bodies. They shape alternative ways of creating, transferring, and transforming archives that not only challenge or counter dominant hegemonic and popular archival practices but, most importantly, exceed them. This article is an attempt to map archives of Rumness that fall through the cracks of the minority’s dominant archival politics. It demonstrates how the minority performs and enacts its archives—what I defined as archival enactment—in order to actively participate in its mnemonic preservation.

While archival practices that focus on the everyday instead of the institutional are not by default decolonial, they can offer insight into archival research methodologies that question the power differential between the researcher and the researched. For Basel Abbas and Ruanne Abou-Rahme, “the vitality that turns the archive into something living is fundamentally connected to a moment of political becoming […] the very act of producing and sharing subjective, horizontal archives is precisely about the instance on and the fight for a living common archive, from the ground up” (2013, pp 353). Archival science methodologies that pay attention to performance both as an object and a method of researching the archive entail the capacity to intensify the “living” moments of enacted “political becomings” in ways that imagine and actualize archival resilience in and through the archival commons.

Many members of my Rum family have been unable to retrieve important documentation, such as birth certificates, necessary for their bureaucratic processes. Challenging the popular assumption that written archival documents remain due to their textuality and materiality, my family’s disappearing documents were untraceable and unretrievable through the state’s archival population management mechanisms. They were present, however, in different forms in the community’s theatre archives and their ephemeral performances that I collected or witnessed during my research. My immediate relatives’ names were mentioned in theatre playbills, posters, and advertisements, or by the members of the Rum community who shared stories relevant to these archives while enacting them. Even though a theatre playbill or an enacted narration of a story cannot replace the lack of access to the official governmental documents, they shape different support systems built of notions of alternative belonging. Therefore, they perform as collective resilience in ways that, in Anne J. Gilliland and Michelle Caswell’s terms, question the “unattainable archive” by imagining and enacting “impossible archival imaginaries” (2016, pp 53). Rum archives render the archival impossibility into an archival actuality through imagination, enactment, and performance. They, thus, underline the necessity and lay the grounds for a performative epistemology of archival research.

## Footnotes

1 All translations are by the author unless otherwise indicated.

2 For more on the discussions regarding the term Rum see Αναστασιάδου and Ντυμόν pp 9–10, Örs pp 3–4, and Alexandris pp 18–19. Alexandris associates the term Rum primarily with the ways the Ottoman Empire and modern Turkey refer to its Greek Orthodox populations and adopts the term Ottoman Greeks or Greeks in English instead. Anastasiadou and Dumont maintain the term Rum in Greek [Ρωμηός] in order to align with the community’s self-identification while maintaining the connections between the Rum minority and the Roman Empire and differentiating them from Turkey’s other Greek-speaking populations. Örs introduces the term Rum Polites in order to refer to the Rums of Istanbul, in Greek Konstantinoupolites or, in short, Polites [Πολίτες] in order to differentiate the community from other Rum populations in Turkey, preserve its bilingualism, and address the community’s connection with the city of Istanbul. Drawing upon the works of Örs and Anastasiadou and Dumont, Ι follow the community’s self-identification, and I use the term Rum in English also in order to indicate that the community’s interests do not always align with the Greek nation-state.

3 For more on Kürkçoğlu’s process visit https://archive.ert.gr/68720/

4 According to handwritten notes retrieved from a personal archive
